# Unveiling the Chemical Composition and Biological Activity of Extracts from the Antarctic Yeast *Dioszegia* sp. AL_105_ and *Bannozyma* sp. AL_104_

**DOI:** 10.3390/molecules31142486

**Published:** 2026-07-16

**Authors:** Snezhana Rusinova-Videva, Maya Margaritova Zaharieva, Dimitrina Zheleva-Dimitrova, Vesela Lozanova, Reneta Gevrenova, Valentin Lozanov, Stefka Nachkova, Dimitar Bojilov, Tsvetislava Kamenova, Hristo Najdenski, Spiro Konstantinov

**Affiliations:** 1 Department of Biotechnology, The Stephan Angeloff Institute of Microbiology—Bulgarian Academy of Sciences (BAS), 139 Ruski Blvd., 4000 Plovdiv, Bulgaria; cvetislava1725@abv.bg; 2 Department of Infectious Microbiology, The Stephan Angeloff Institute of Microbiology—Bulgarian Academy of Sciences (BAS), 1113 Sofia, Bulgaria; zaharieva@yahoo.com (M.M.Z.); hnajdenski@gmail.com (H.N.); 3 Department of Pharmacognosy and Pharmaceutical Botany, Faculty of Pharmacy, Medical University of Sofia, 2 Dunav Str., 1000 Sofia, Bulgaria; dimizheleva@gmail.com (D.Z.-D.); rgevrenova@gmail.com (R.G.); 4 Department of Medical Chemistry and Biochemistry, Faculty of Medicine, Medical University of Sofia, 2 Zdrave Str., 1000 Sofia, Bulgaria; vlozanova@medfac.mu-sofia.bg (V.L.); vlozanov@medfac.mu-sofia.bg (V.L.); 5 Department of Analytical Chemistry and Computer Chemistry, University of Plovdiv “Paisii Hilendarski”, 4000 Plovdiv, Bulgaria; stefka_nachkova@abv.bg; 6 Department of Organic Chemistry, University of Plovdiv “Paisii Hilendarski”, 4000 Plovdiv, Bulgaria; mbojilov@gmail.com; 7 Department of Pharmacology, Pharmacotherapy and Toxicology, Faculty of Pharmacy, Medical University of Sofia, 2 Dunav Str., 1000 Sofia, Bulgaria; konstantinov.spiromihaylov@gmail.com

**Keywords:** Antarctic yeasts, metabolites, biological activity

## Abstract

The extreme Antarctic environment offers unique living conditions. The organisms inhabiting Antarctica have a specific metabolism through which they overcome the challenges of large temperature amplitudes, long periods of light or darkness, and intense UV radiation. Microorganisms are ubiquitous in Antarctica and have been the subject of intensive research in recent decades. In this manuscript, two yeast species were isolated from Antarctic habitats and identified as *Dioszegia* sp. AL_105_ (AL_105_) and *Bannozyma* sp. AL_104_ (AL_104_). The cultivation of these two species under submerged conditions were evaluated. The strain AL_104_ managed to accumulate over 7 g/L biomass for 96 h. For the first time, the cytotoxic activity of the obtained yeast extracts on malignant cell lines has been studied. The obtained methanol and acetone extracts showed different cytotoxicity when used to treat different malignant cell lines, with maximal values of IC_50_ = 113 ± 8.2 established for the HUT-78 line with AL_104_. The methanol extracts of both yeast species exhibited low antibacterial activity against *Staphyloccocus aureus*, MRSA, and *Escherichia coli*. Additionally, the methanol extract of AL_105_ inhibited the biofilm formation of MRSA to a higher extent than the methanol extract from AL_104_. Antioxidant activities were determined by using HPSA, HRSA, and NOSA tests. The chemical characterization of the extracts showed the presence of various lipids—CoQ_10_, triglycerides, phosphatidylethanolamine, and ceramide. It is likely that the interaction between the different bioactive molecules can explain the observed cytotoxicity. The current new studies have proven that Antarctic yeasts are a potential source of biologically active molecules with the potential for future biomedical research.

## 1. Introduction

Although Antarctica is the geographical region that represents the largest polar territory, it is the least explored one. As one of the most extreme environments, the Antarctic desert requires the presence of some key survival mechanisms in these microorganisms in order for them to develop. They have developed a set of structural and functional adaptations to ensure their existence and overcome the main attribute of Antarctica—significantly low temperatures. Antarctic yeasts are widely spread and have been frequently studied by various researchers due to their ability to adapt to a psychrophilic lifestyle. Some of these adaptations are related to changes in the fluidity of cell membranes, the synthesis of pigments as a response to intense UV radiation, or the synthesis of a polymeric coating against excessive dryness. Such features are summarized as life “at the ecological edge” [1]. The presence of a specific metabolism in the insufficiently studied Antarctic yeasts suggests the existence of new unexplored biomolecules. This has resulted in significant scientific interest in these unique microorganisms, searching for and identifying new metabolites and exploring their application in various practical fields.

Some of the rare yeast genera found in Antarctica are *Dioszegia* and *Bannozyma*. The limited research on these yeasts directed our interest towards investigating their biosynthetic potential and possible biological activities. Species such as *Dioszegia* sp. or *D. fristingensis* have been described as producers of a variety of enzymes [2,3,4]. Other bioactive substances that the genus *Dioszegia* contains are peptides. Five peptides rich in proline and glycine (24% and 29%, respectively) have been found, their chain length ranging from 12 to 17 amino acids. The hypotheses suggest that these psychrophilic yeast-specific peptides play a role in cellular signaling [5]. There are ongoing studies on their localization in the extracellular environment and their function inside and outside the cell [5,6]. Other bioactive molecules identified in *Dioszegia* species are the mycosporins. It is believed that they aid the species in adapting to the harsh Antarctic conditions (photoprotective properties), either alone or in combination with pigments [7,8,9]. *Bannozyma yamatoana* and *Bannozyma* sp. have also been studied as enzyme producers [10].

The screening of the Antarctic yeasts for the attainment of natural products and molecules that have potential biotechnological applications is of great importance. Often, studies aimed to obtain enzymes by Antarctic yeasts. The proteases, cellulases, esterases, lipases, amylases, and pectinases obtained from Antarctic producers were proposed as useful for biotechnological applications [10]. Lipase A and lipase B produced by Antarctic *Candida antarctica* had a high catalytic potential and have been widely used in the biological, chemical, and food industries [11]. To obtain valuable metabolites, different types of stress on producers have been evaluated. In addition to demonstrating an adaptive capacity in Antarctic yeast, stress can also be used to produce new bioactive molecules [12]. After an analysis of the metabolic profile of *Rhodotorula mucilaginosa* AN5 in the presence of chemical-stress-inducing agents, new metabolites were identified [13]. Metabolic changes were also demonstrated during temperature stress in Antarctic yeast, accompanied by an increased exopolysaccharide production [14]. *Rhodotorula glutinis* has been identified as yeasts with great industrial potential for the synthesis of β-carotene, torulene, torularhodin, and other carotenoids. Their application in the cosmetics, pharmaceuticals, and food industry, as additives in livestock, fish, and crustaceans feed, are due to their health-promoting properties [15].

The presence of high-value biomolecules in Antarctic yeast species directs our interest. This study represents the first investigation of the cellular growth in submerged cultivation, and the biological activities of Antarctic yeast (AL_104_ and AL_105_) extracts against cancer cells and prokaryotic pathogens, as well as the antioxidant capabilities of these extracts. That is why uncovering the metabolic cellular composition is of great importance for characterizing new molecules and their possible effects.

## 2. Results

**Figure 1:**
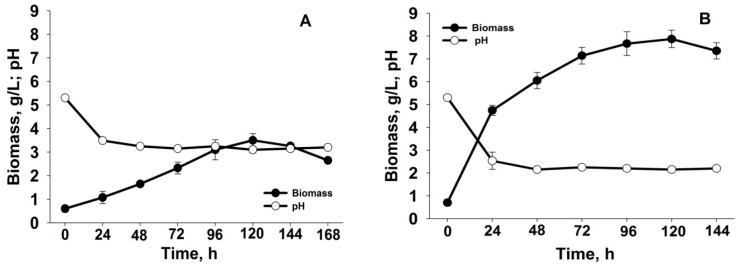
Cell growth dynamics and pH monitoring of culture by (**A**) *Dioszegia* sp. AL_105_, and (**B**) *Bannozyma* sp. AL_104_, at 220 rpm, 22 °C, 168 and 144 h.

The yeast isolates were identified by a sequence analysis of their genomic ITS regions. The data analysis identified the first isolate as *Dioszegia* sp. AL_105_ and the second isolate as *Bannozyma* sp. AL_104_. The different coloring of each species was visible in the photographic material. *Dioszegia* sp. AL_105_ was orange, while *Bannozyma* sp. AL_104_ was beige in color (Figure S1). The growth capabilities of these yeasts were investigated from a biotechnological point of view. The cellular growth of both strains was examined in terms of their dynamics (Figure 1). The amount of biomass was monitored in depth, with samples taken every 24 h. The optimal biomass accumulation in both strains occurred between 96 and 120 h, which shows that the duration of the process could be reduced to 120 h. The transition from the lag phase to the exponential phase was graphically represented, showing a faster rate in *Bannozyma* sp. AL_104_, taking less than 20 h (Figure 1B).

For *Dioszegia* sp. AL_105_, this process is longer and proceeds at a slower rate of at least 24 to 48 h. The optimal biomass accumulation achieved was between 3 g/L and 4 g/L for *Dioszegia* sp. AL_105_, while the values for *Bannozyma* sp. AL_104_ reached over 7 g/L. The death phase occurred after 120 or 144 h for both producers. In parallel with the monitoring of cell growth, a change in pH values was also recorded. Regarding pH, an expected decrease in the values of the curve trend was observed during the fermentation, with *Bannozyma* sp. AL_104_ reaching lower values than *Dioszegia* sp. AL_105_. The initial pH values of the culture medium were 5.3, while, after 24 h, in AL_105_, these values reached pH 3.3 and, in AL_104_, they decreased to pH 2.1. Antarctic cells continued to grow even at a low pH (Figure 1 and Table S1).

The obtained methanol extracts by AL_104_ and AL_105_ were in the amounts of 0.153 g/g LB and 0.25 g/g LB, respectively, while the acetone extracts were in the amounts of 0.06 g/g LB and 0.09 g/g LB.

The tumor growth inhibiting the properties of methanol and acetone yeast extracts were evaluated in in vitro studies on human cutaneous T-cell lymphoma (CTCL) and urothelial cancer cells (Table 1). Non-tumorigenic murine fibroblasts were used in the evaluation of the in vitro cytotoxicity. It is noteworthy that none of the extracts exerted any antiproliferative activity on the normal murine fibroblast cells CCL-1. This is an indication of selective antineoplastic activity against the malignant cell lines used. CTCL cell lines were characterized with a higher sensitivity to the tested yeast extracts, whereby the proliferation of the Sézary-syndrome-derived HuT-78 cells was inhibited with lower IC_50_ concentrations than the mycosis-fungoides-derived MJ cell line. Both urothelial cancer cell lines were less sensitive to treatment with the yeast extracts than the lymphoma cells. Interestingly, the *Dioszegia* sp. AL_105_ and *Bannozyma* sp. AL_104_ methanol extracts were more active (IC_50_ concentration below the tested limit of 400 µg/mL) against the CAL-29 cell line which originates from a human high-grade, invasive bladder tumor found to reach in.

The results from the BMD test are presented in Table 2. Both extracts exhibited low antibacterial activity against *Staphyloccocus aureus*, MRSA, and *Escherichia coli*. The MICs on all strains were 10 mg/mL for the *Bannozyma* sp. AL_104_. The *Dioszegia* sp. AL_105_ extract exhibited stronger activity on the Gram-positive strains—the determined MIC was 5 mg/mL. The established activity was bacteriostatic in these concentrations, with the MBC being above 10 mg/mL.

Additionally, the potential of both extracts to inhibit the biofilm formation capacity of the MRSA strain was determined in concentrations equal or below the estimated MIC values. As visualized on the micrographs in Table 3, a concentration-dependent inhibitory effect was observed. The more active extract (from *Dioszegia* sp. AL_105_) inhibited the biofilm formation of MRSA to a higher extent than the methanol extract from *Bannozyma* sp. AL_104_. At a concentration of 2 × MIC, it inhibited the ability of the bacteria to attach to the target surface by 90%. This effect decreased directly proportionally to the applied concentration. The median biofilm inhibitory concentration is approx. 2.5 mg/mL. (1/2 xMIC). The statistical evaluation of the absorbance results presented on the graph is given in Supplementary Table S2.

The investigated methanol extracts of *Dioszegia* sp. AL_105_ and *Bannozyma* sp. AL_104_ showed antioxidant activity, assessed by HPSA, HRSA, and NOSA tests (Table 4). *Dioszegia* sp. AL_105_ demonstrated higher antioxidant activity than *Bannozyma* sp. AL_104_, having a higher value of µM QE/gLB and a lower IC_50_, indicating a better ability to neutralize hydrogen peroxide and ^•^OH radicals. In addition, the same extract was also characterized by good activity against NO^•^.

The preliminary LC-MS screening analyses of the tested extracts in negative and positive ion mode revealed the presence of 20 compounds, including 5 organic acids and sugars, 2 fatty acids, 3 amino acids, 3 terpenes and sterols, 4 lysophosphatidylcholines (LysoPCs), and 3 apocarotenoids (Table 5). The annotation/dereplication of the metabolites was carried out based on the MS and MS/MS accurate masses, retention times, fragmentation patterns, relative ion abundance, and comparison with literature data (Table S3) [16,17,18]. Identification confidence levels were estimated according to Çiçek (2024) [19], and were as follows: tentative identification based on libraries, model compounds, etc. (D); relatively reliable evidence (D1); and relatively poor evidence (D2).

The lipid composition of the studied strains was presented in Table 6.

The heat map clearly visualized metabolites that were found in a higher percentage (red spot) versus metabolites that were less frequently found in the extracts (blue spot). If we look at the first two columns for methanolic and acetone extracts of *Doiszegia* sp., it could be summarized that the last 20 of the metabolites listed on the right predominate in it (red spots), while, in the extracts of *Bannozyma* sp., there was a more complex configuration of red spots. The first 20 metabolites in the right column are more likely to occur in the acetone extract, while 9 of those in the middle of the metabolite column were more common in the methanol extract of the species.

## 3. Discussion

The newly isolated species belong taxonomically to the kingdom Fungi, subkingdom Basidiomycota, with their differences being due to the different classes to which they belong. *Dioszegia* sp. AL_105_ belongs to the class *Tremellomycetes*, family *Bulleribasidiaceae*, genus *Dioszegia*. *Bannozyma* sp. AL_104_ is a representative of class *Microbotriomycetes,* family *Chrysozymaceae*, genus *Bannozyma*. Yeasts of the genus *Dioszegia* can be found in both temperate and polar regions. They were discovered in soil samples and their phylogenetic affiliation to *Tremellomycetes*, *Agaricomycotina* was proven in 2010 by Conell et al. [20]. In 2013, they were isolated from an Antarctic lake [21]. The Antarctic species of *Dioszegia* demonstrated additional enzymatic activities. For *Dioszegia* sp., these included amylase, esterase, and pectinase, while amylase, cellulase, lipase, pectinase, chitinase, and xylanase were produced by *D. fristingensis* [2]. In one of the psychrotolerant species, amylase activity with an optimum temperature of 37 °C has been described [3]. The practical significance of these enzymes makes *Dioszegia* sp. a reliable producer, and the low-temperature activity in some of them at 4 °C and 10 °C makes them unique for practical applications [4]. Species of the genus *Bannozyma* are not widely spread, with only three reports of such yeast species available in the literature. They are described as inhabiting the surface of Antarctic lichens and snow [22]. The genus *Bannozyma* was classified within the *Chrysozymaceae* family, class *Microbotryomycetes* in 2015 [23]. The studies on these species were focused on enzyme synthesis. *B. yamatoana* demonstrated pectinase and protease activity, while other representatives of *Bannozyma* sp. synthesized amylase, cellulase, esterase, lipase, pectinase, and protease [10]. Both yeast species are not well-studied but are of interest regarding their cellular growth capabilities and behavior under submerged cultivation, as well as the biological activity of the biomolecules synthesized. The data showed that cellular growth followed the curve visualized in Figure 1. The optimal biomass accumulation for *Bannozyma* sp. AL_104_ was observed at about 120 h and it had the highest values compared to other Antarctic yeasts cultivated under the same conditions [24,25], which is encouraging for the future scaling of the process. The advantage of the conducted cultivation was the natural pH reduction, which helps prevent contamination of the process.

The effects of the methanolic and acetone extracts of the two Antarctic yeast strains on various malignant cell lines were demonstrated for the first time by determining the mean inhibitory concentrations. Taken together, the experimental data summarized in Table 1 show that the studied Antarctic yeast extracts possess a moderate antineoplastic potential with a distinct antitumor spectrum of activity. However, the antiproliferative potential of the extracts could be explored in future investigations, having in mind the specificity of the neoplastic diseases which were targeted by using the selected cell lines. The MJ cell line is representative for mycosis fungoides which is cutaneous T-cell lymphoma (CTCL). The treatment modalities of CTCL also include, besides the systematic therapy, a number of skin-directed therapeutic schemas (retinoids, local corticosteroids, etc.) for limited skin disease and patients with favorable overall survival [26]. In addition, for first-line therapy for erythrodermic mycosis fungoides, extracorporeal photopheresis is considered, which is characterized by an excellent side effect profile and moderate efficacy [27]. In this regard, the application of a higher concentration of bioactive extracts/compounds such as the yeast extracts could be investigated in future in in vitro and in vivo studies. The same approach related to local therapeutic schemas applies for bladder carcinoma. The cell lines T-24 and Cal-29 are representative for bladder carcinoma whose therapy includes local treatment with epirubicine in high doses (0.5–1 mg/mL) [28]. Therefore, the authors consider that the active concentrations of the tested yeast extracts are relevant to the current clinical treatment schemas and have the potential for pharmacological development, especially in combination with clinically applied chemotherapeutics which could be a subject for future investigations.

The antibacterial activity of the yeast extracts was tested on one reference Gram-positive (*Staphylococcus aureus*) and one reference Gram-negative (*Escherichia coli*) strain. The inhibition of the bacterial growth was achieved after incubation with rather high concentrations of the extracts (5 mg/mL for the *Dioszegia* sp. AL_105_ extract and 10 mg/mL for the *Bannozyma* sp. AL_104_ extract) which points to a low antibacterial potential excepting the treatment of local cutaneous infections. The observed inhibition effect of the *Dioszegia* sp. AL_105_ extract on the formation of the MRSA biofilm supports this possibility for further pharmacological studies. The yeast extracts represent a complex mixture of different metabolites and their moderate activity could be useful in combination with clinically applied antibiotics in order to potentiate their activity which remains a perspective for future studies.

Cells produce reactive oxygen species (ROS) and reactive nitrogen species (RNS) as natural byproducts of cellular metabolism. Major ROS include superoxide anion (O_2_^•−^), hydrogen peroxide (H_2_O_2_), and hydroxyl radical (^•^OH), whereas nitric oxide (NO^•^) and peroxynitrite (ONOO^−^) are among the principal reactive nitrogen species (RNS). Under physiological conditions, ROS and RNS participate in cellular signaling and homeostatic regulation; however, their excessive accumulation can lead to oxidative and nitrosative stress, resulting in cellular damage and dysfunction. These molecules assist in cell signaling and immune defense during normal physiological states. The excessive buildup of these molecules results in oxidative and nitrosative stress which damages DNA along with lipids and proteins while facilitating the emergence of neurodegenerative diseases and cardiovascular and inflammatory conditions. Antioxidants and enzyme systems play an important role in controlling RNS and NOS to sustain cellular homeostasis while preventing oxidative cellular damage [29,30,31,32].

The antioxidant activity assessment demonstrated notable differences between the investigated yeast isolates (Table 4). *Dioszegia* sp. AL_105_ exhibited a higher antioxidant capacity than *Bannozyma* sp. AL_104_, particularly in hydrogen peroxide scavenging activity (HPSA) and hydroxyl radical scavenging activity (HRSA), indicating a greater ability to neutralize reactive oxygen species. Although the antioxidant effects of both microbial extracts were significantly lower than those of quercetin, this outcome was expected due to the well-established and potent antioxidant properties of this natural reference compound.

Despite their lower activity compared with quercetin, the microbial extracts displayed considerable antioxidant potential, suggesting the presence of bioactive metabolites capable of mitigating oxidative stress. These findings highlight the potential of yeast-derived extracts as natural sources of antioxidant compounds with possible applications in pharmaceutical, nutraceutical, and anti-inflammatory formulations. Overall, *Dioszegia* sp. AL_105_ emerged as the more promising strain, demonstrating superior free radical scavenging activity and warranting further investigation for the isolation and characterization of its active constituents.

**Figure 2:**
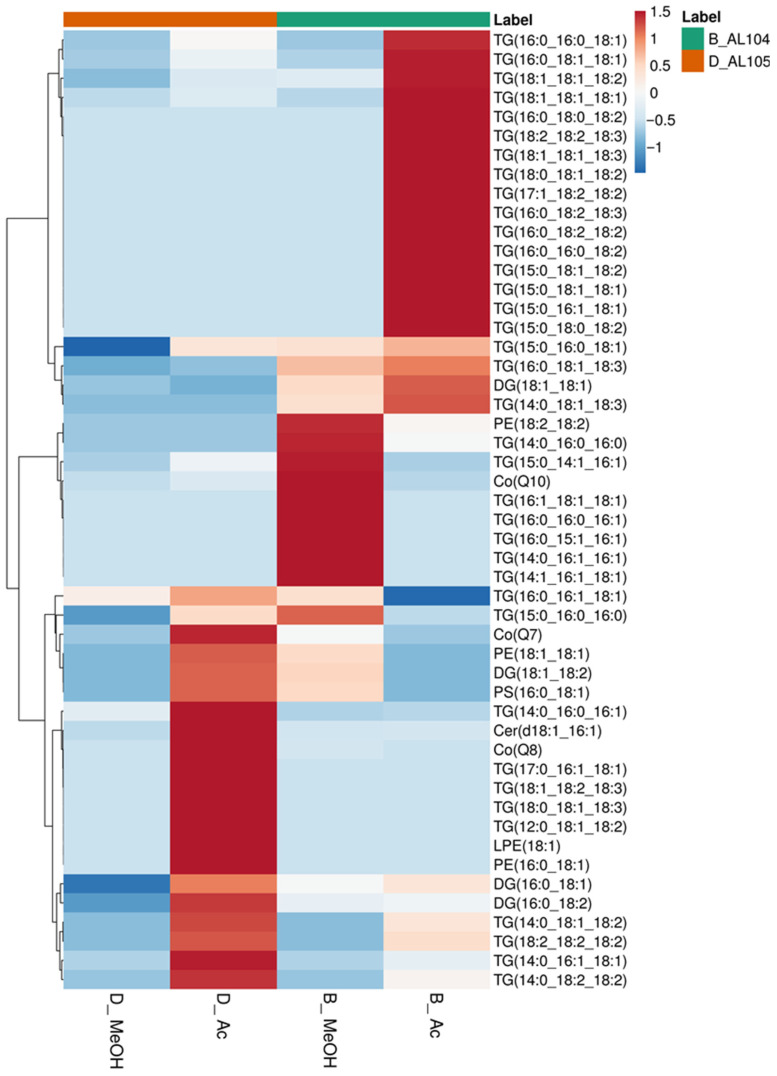
Heatmap of the top 50 lipids with maximal total intensity difference between classes. (Legend: B_Ac-*Bannozyma* sp. AL_104_ acetone, B_MeOH-*Bannozyma* sp. AL_104_ methanol, D_Ac-*Dioszegia* sp. AL_105_ acetone, D_MeOH-*Dioszegia* sp. AL_105_ methanol).

In general, the lowest amount of lipids was extracted from the biomass of strain *Dioszegia* sp. AL_105_ with methanol. For the remaining extracts, the number of identified metabolites was comparable; however, for about half of their total count, they were found in only one of the extracts. Figure 2 of the fifty most distinguishing lipids shows which of them are more abundant for each extract. It can be seen that CoQ_10_ with some triglycerides and phosphatidylethanolamine PE (18:2_18:2) were extracted in the largest amount from strain *Bannozyma* sp. AL_104_ with methanol. The other CoQ_7_ and CoQ_8_ were found in higher quantities in the acetone extract of strain *Dioszegia* sp. AL_105_ than in the methanol solution of *Bannozyma* sp. AL_104_, along with ceramide (d18:1_16:1) which is absent in the *Dioszegia* sp. AL_105_ methanol extract only. Two of the several known effects—anti-inflammatory and antibacterial effects—are valuable for the cosmetic applications of azelaic acid (Table 5). This metabolite is described as meeting Lipinski’s requirements with the possibility of pharmacological application. Azelaic acid was identified in Antarctic lichens [33], but has not been reported as a metabolite from Antarctic yeasts. The reported amino acids leucine and phenylalanine are also described at *Sporobolomyces roseus* AL_103_ [14]. The dihydroactinidiolide was identified in all extracts except the acetone extract of AL_104_. It belongs to the group of terpenes and is known for its antioxidant, antibacterial, and anticancer effects. It is known to be a breakdown product of the β,β-carotene molecule [34]. The data showed the good proapoptotic activity of essential oils containing dihydroactinidiolide in the treatment of malignant melanoma cells [35]. Some metabolites belonging to the carotenoid or apo-carotenoid group have been identified. These were astaxanthin, β-ionone, β-cyclocitral, and β-apo-13-carotenone. The production of higher amounts of carotenoids and ergosterol is considered to be a response to strong UV radiation. As a species, *Dioszegia* has been identified as a producer of high amounts of carotenoids [36]. Due to the characteristic high levels of UV radiation in the polar regions, metabolites belonging to the isoprenoid group have been recognized in Antarctic yeasts. It is believed that they can repair cellular damage caused by ROS resulting from the radiation exposure. Some species have been found to produce torularodine, torulene, γ-carotene, β-carotene, and astaxanthin [9]. In the Antarctic producer *Sporobolomyces salmonicolor* AL_1_, the antioxidant activity of ergosterol, torularhodin, torulene, β-carotene, and CoQ_10_ in comparison with trolox was confirmed, and their photoprotective function was established after the UVA absorption in a model emulsion [37]. In the microbial biosynthesis of isoprenoids, obtaining the substances from natural sources is very important. Studies on torulene and torularhodine have demonstrated their protective role as part of the diet in rats in dimethylnitrosamine-induced neoplastic liver changes and additionally in the significant inhibition of prostate cancer [38]. Scientific efforts to achieve better yields of carotenoids have focused on changing the abiotic environmental factors and seeking rational methods of genetic engineering [38,39,40]. Ceramide (Table 6) was part of the lipid composition of the acetone extracts. It was discovered as part of a larger disaccharide molecule with immunogenic properties in Antarctic krill [41]. It is a sphingolipid metabolite and has been implicated in signal transduction that affects cellular life [42]. Ceramide is believed to be an important factor in inducing apoptosis and the subsequent autophagy of cancer cells. Ceramide analogs have even been developed to imitate ceramide-like apoptosis. Based on them, the development of anticancer drugs is optimized [43]. Phosphatidylserine and phosphatidylinositol, which are known to serve as signaling molecules in the processes of programmed cell death repair [44], were detected in three of the extracts.

## 4. Materials and Methods

### 4.1. Microorganisms and Growth Conditions

The isolate AL_105_ was isolated from penguin feathers on rocks in a coastal area on Perunika region (62°38′6.6″ S, 60° 21′14.52″ W).

The isolate AL_104_ was isolated from soil on rocks with the following coordinates: (62°38′46.5″ S, 60°22′12.42″ W).

The isolates grew at 4 °C on malt agar after approximately 14–20 days. Genomic DNA was purified from the strains, and sequencing of the ITS1 and ITS4 regions was performed. The isolates were identified by BLAST analysis (https://blast.ncbi.nlm.nih.gov/Blast.cgi?PROGRAM=blastn&PAGE_TYPE=BlastSearch&LINK_LOC=blasthome, accessed on 10 July 2026). The sequences were submitted in NCBI, with isolate AL_105_ identified as *Dioszegia* sp. AL_105_ and assigned NCBI accession number PQ351404, while isolate AL_104_ was identified as *Bannozyma* sp. AL_104_ with accession number PQ363140.

The submerged cultivations were carried out in 500 mL flasks with 50 mL medium. The cultural medium contained the following (in g/L): sucrose 40.0; yeast extract, 1.0; (NH_4_)_2_SO_4_, 2.5; KH_2_PO_4_, 1.0; MgSO_4_.7H_2_O, 0.5; NaCl, 0.01; and CaCl_2_.2H_2_O, 0.01. The inoculum culture was 10% of cultural medium and was carried out at 22 °C, on a rotary shaker with 220 rpm mechanical stirring for 48 h. Cultivation process was performed at 22 °C on a rotary shaker with 220 rpm mechanical stirring for 120 h.

### 4.2. Biomass Production and Preparation of Extracts

Cells were pelleted by centrifugation at 6000× *g* for 30 min, prior to dry weight determination. The dry weights of the samples were determined by drying at 105 °C until constant weight. The standard deviation for dry weight samples was 0.1 to 0.3 g/L.

Lyophilized biomass was used for extraction. Then, 1 g of dried biomass was added to extraction reagent (methanol (100%) or acetone (100%)) in a ratio of 1:20. The suspensions were subjected to double ultrasonic extractions for 20 min. The resulting suspension after each cell disintegration was filtered through a filter. To obtain dry extracts, the solutions were dried by vacuum-evaporation.

Sigma Plot 2001 was used for statistical processing of the results and graphical layout.

### 4.3. Cell Lines and Culture Conditions

The cell lines used for evaluation of antineoplastic activity were as follows: CCL-1, HUT-78, MJ, T-24, and CAL-29. CCL-1TM, HuT-78, and MJ were purchased from the American Type Culture Collection (ATCC, Manassas, VA, USA) and cultured in culture medium MEM supplemented with 10% heat-inactivated horse serum (Capricorn GmbH, Ebsdorfergrund, Germany) and 2 mM L-glutamine (Capricorn GmbH, Ebsdorfergrund, Germany). T-24 and Cal-29 were obtained from DSMZ (Braunschweig, Germany) and cultured in medium DMEM (Capricorn GmbH, Ebsdorfergrund, Germany) with 10% heat-inactivated fetal bovine serum (Capricorn GmbH, Ebsdorfergrund, Germany) and 4 mM L-glutamine (Capricorn GmbH, Ebsdorfergrund, Germany). Pen/Strep (concentration of penicillin G sodium 105 Units/L and of streptomycin sulphate 100 mg/L) was added to both culture media. The cell lines were maintained as an adherent monolayer in a log phase. Cells were maintained under sterile conditions in a CO_2_ incubator (Panasonic MCO-18AC, Osaka, Japan) at 37 °C, humidified atmosphere, and supply of 5% CO_2_. The cell lines were sub-cultivated 2 times per week following the protocols of the relevant biobank. The adherent cells were detached with 0.25% trypsin after washing the cell monolayer with PBS containing 0.53 mM EDTA. Thereafter, cells were re-suspended in culture medium in order to deactivate the enzyme and centrifuged for 5 min at 300× *g*. The supernatant was removed and cells were re-suspended and sub-cultivated in fresh medium at seeding density 1.0 × 10^4^ cells/cm^2^.

### 4.4. Cytotoxicity Assessment (MTT-Dye Reduction Assay) of Cell Lines

The antiproliferative activity of both extracts was evaluated by using the MTT test following the protocol in Annex C, ISO 10993-5 [45,46]. Briefly, 100 µL of cell suspension with density 1 × 10^5^ mL^−1^ for the adherent cells and 3 × 10^5^ mL^−1^ for the suspension cell lines were seeded per well in 96-well plates with flat bottom. The cells were incubated for 24 h in order to enter the log phase of the cell growth and were exposed to different concentrations of the yeast fractions. Each sample was repeated four times. The survived cell fraction was measured after 72 h of incubation by using the MTT dye in a final concentration of 0.5 mg/m. The formazan crystals were dissolved in 2-propanol supplemented with 5% formic acid. The pure solvent was used as a blank. Untreated cells served as negative control. The absorption was measured at λ = 550 nm (reference filter 690 nm) on a microplate reader ELx800 (BioTek Instruments, Inc., Winooski, VT, USA). The median inhibitory (IC_50_) concentrations were calculated in the GraphPad Prism software (Version 6.00, for Windows, GraphPad Software, La Jolla, CA, USA) using the mathematical model [log(inhibitor) vs. normalized response—variable slope]:

(1) $$Y=\frac{100}{1+10^{(log{IC}_{50}-X)\times hillslope}}.$$

The statistical evaluation was performed with the same program based on the mathematical model used with R_2_ ≥ 0.95.

### 4.5. Bacterial Strains and Growth Conditions

Two referent bacterial strains (one Gram-positive and one Gram-negative) were used in this study for the evaluation of antimicrobial activity: *Staphylococcus aureus* ATCC 29213, and *Escherichia coli* 25922. The strains were purchased from the American Type Culture Collection, Virginia, NV, USA and are recommended for such studies in ISO Standard 20776/1-2006 (ISO 20776/1-2006). For evaluation of the antibiofilm activity, the methicillin-resistant strain *S. aureus*—MRSA #8327 (Bulgarian Culture Collection) was used. The bacterial cultures were grown in BHI broth (Himedia, Mombai, India) at 37 °C (Thermostat Memmert GmbH, UN30, Schwabach, Germany). The same medium was used for determination of the minimal inhibitory and bactericidal concentrations according to the broth microdilution test (BMD) as described in ISO 20776/1-2006.

### 4.6. Broth Microdilution Test and Redox Activity

The BMD test was applied according to ISO 20776/1-2006 [47] for estimation of the minimal inhibitory concentrations of both tested extracts. The extracts were dissolved in 70% ethanol in situ to concentration of 50 mg/mL. From this solution, twofold serial dilutions in concentrations ranging between 10 and 0.02 mg/mL were prepared in triplicate in 96-well plates in a final volume of 50 µL/well. An equivalent volume bacterial suspension (5 × 10^5^ CFU/mL) from the tested bacterial strain (listed in the previous subsection) was added to each well. Thereafter, the plates were incubated overnight at 37 °C and the result was read visually on the next day following the recommendations in ISO 20776/1-2006, as minimal inhibitory concentration (MIC) was determined as the lowest concentration that led to inhibition of visible bacterial growth. Two clinically approved antibiotics were selected as reference drugs for positive controls—gentamycin and penicillin (Gibco, Life Technologies Ltd., Paisley, UK). The negative control was phosphate-buffered saline. BHI broth without extracts, antibiotics, or PBS was incubated in parallel to the samples as control for lack of contamination. They were applied in concentrations of 0.008 to 4 mg/L. The results were analyzed according to the recommendations of EUCAST (European Committee on Antimicrobial Susceptibility Testing).

The metabolic activity of the treated bacteria and the controls was measured on an ELISA reader (Absorbance Microplate Reader Lx800, Bio-Tek Instruments Inc., Winooski, VT, USA) after determination of the MICs. The redox activity was measured colorimetrically based on the product of the MTT dye (3-(4,5-dimethylthiazolyl-2)-2,5-diphenyltetrazolium bromide, #M2128, Sigma^®^ Life Science, Steinheim, Germany, which was added to each well to a final concentration of 0.5 mg/mL [23]. The MTT is reduced by the membrane-located bacterial enzyme NADH: ubiquinone reductase (H+-translocation) to the water-insoluble compound formazan. The formazan crystals were dissolved in 2-propanol containing 5% formic acid and the absorbance was measured at 550 nm (referent filter 690 nm). A mixture of BHI, MTT, and the organic solvent served as a blank solution.

### 4.7. Biofilm Formation Assay

The biofilm formation assay was carried out following the protocol of Stepanovic et al. (2007) [48]. For this aim, twofold serial dilutions of the yeast extracts were prepared in triplicate in flat-bottom 96-well polystyrene tissue culture plates. The concentrations ranged between 10 and 2.5 mg/mL in BHI broth supplemented with 2% D-Glucose (*w*/*v*) to a final volume of 100 µL/well. To each well was added an equivalent volume of MRSA bacterial suspension with cell density of 5 × 10^5^ CFU/mL. After 24 h of incubation at 37 °C, the culture medium was discarded and the attached bacterial cells were washed three times with PBS (150 µL/well), fixed in methanol (200 µL/well, 15 min), and stained with 0.1% crystal violet (50 µL/well, 5 min). The dye solution was removed and the wells were washed with tap water. The air-dried samples were documented under an inverted light microscope. For the quantitative measurement, 160 μL of 33% acetic acid was added to each well and the absorbance was measured at λ = 550 nm. The values were calculated as % of the untreated control.

### 4.8. Antioxidant Activity

#### 4.8.1. Hydrogen Peroxide Scavenging Activity (HPSA)

The Manolov et al. approach was used to evaluate a capacity to scavenge hydrogen peroxide [49]. A 43 mM solution of H_2_O_2_ was prepared in potassium phosphate buffer solution (0.2 M, pH 7.4). The analysis of the samples was carried out as follows: in test tubes, 0.6 mL H_2_O_2_ (43 mM), 1 mL sample/standard with different concentrations (20–1000 µg/mL), and 2.4 mL potassium phosphate buffer solution were mixed. The mixture was stirred and incubated in the dark for 10 min at 37 °C. Absorbance was measured at 230 nm with a spectrophotometer (Camspec M508, Leeds, UK) against a blank solution containing phosphate buffer and H_2_O_2_ without the sample. The quercetin was used as standard. The percentage HPSA of the samples was evaluated by comparing with a blank sample and calculated using the following formula:

(2) $$I,\%(HPSA)=\left[\frac{A_{blank}-(A_{TS}-A_{CS})}{A_{blank}}\right]\times 100$$

where *A_blank_* is the absorbance of the blank sample, *A_CS_* is the absorbance of the control sample, and *A_TS_* is the absorbance of the test sample. The antioxidant activity results are presented as micromoles quercetin equivalent per gram of lyophilized biomass (µM QE/gLB) and as IC_50_.

#### 4.8.2. Hydroxyl Radical Scavenging Activity (HRSA)

Hydroxyl radical scavenging activities of various yeast extracts were determined according to the method described by Guo [50]. The scavenging of hydroxyl radicals was done as follows: 0.3 mL sodium salicylate (20 mM), 1 mL FeSO_4_ (1.5 mM), 1 mL sample/standard with different concentrations (20–1000 µg/mL), and 0.7 mL H_2_O_2_ (6 mM). They were mixed immediately, followed by incubation of the reaction tubes in a 37 °C water bath for 1 h. The absorbance values of the mixtures were determined at 510 nm against a blank. We used a standard with proven high antioxidant activity such as quercetin. The hydroxyl radical-scavenging ability was calculated as follows:

(3) $$I,\%(HRSA)=\left[\frac{A_{blank}-A_{sample}}{A_{blank}}\right]\times 100$$

where *A_blank_* is the absorbance without samples and *A_sample_* the absorbance in the presence of the samples. The antioxidant activity results are presented as micromoles quercetin equivalent per gram of lyophilized biomass (µM QE/g LB) and as IC_50_.

#### 4.8.3. Nitric Oxide Scavenging Activity (NOSA)

NO inhibition was measured according to the method of Marcocci [51]. Sodium nitroprusside (SNP) was used as the source for generating NO. The SNP solution (5 mM) was prepared in phosphate-buffered saline (PBS, 0.2 M, pH 7.4). Briefly, the reaction mixture containing 0.5 mL SNP (5 mM), and 1 mL sample/standard with different concentration (15–1000 μg/mL) was incubated at 25 °C for 180 min. At the end of the reaction time, an equal amount of Griess reagent (1% sulfanilamide in 2% phosphoric acid and 0.1% naphthylethylenediamine dihydrochloride) was added. The absorbance of the chromophore (purple azo dye) formed during the diazotization of nitrite ions with sulfanilamide and subsequent coupling with naphthylethylenediamine dihydrochloride was measured at 546 nm. Quercetin was used as standard. The NO uptake capacity was calculated as follows:

(4) $$I,\%(NOSA)=\left[\frac{A_{blank}-A_{sample}}{A_{blank}}\right]\times 100$$

where *A_blank_* is the absorbance of a blank and *A_sample_* is the absorbance of the test sample. The antioxidant activity results are presented as IC_50_.

All assays were carried out in triplicate, and data are presented as mean ± standard deviation (SD). Statistical analyses were performed using GraphPad Prism (version 10.4.0) and Microsoft Excel. Statistical significance was defined at a threshold of *p* < 0.05. The half-maximal inhibitory concentration (IC_50_) values were calculated from three independent determinations and expressed as mean values.

### 4.9. Liquid Chromatography–Mass Spectrometry Profiling (LC-MS)

The analyses were carried out on Orbitrap IQ-X hybrid mass spectrometer (ThermoScientific Co., Waltham, MA, USA) equipped with a HESI^®^ (heated electrospray ionisation) module and Vanquish Ultra High-Performance Liquid Chromatography (UHPLC) system (ThermoScientific Co., Waltham, MA, USA)

The chromatographic separations of the analyzed compounds were achieved on Nucleodur Isis C18 (2.1 × 150 mm, 3 µm) analytical column (Macherey-Nagel, Düren, Germany). using gradient elution at 300 µL/min flow rate. The used eluents were as follows: A—mixture of water/acetonitrile (4/6, *v*/*v*) containing 10 mM ammonium formate, 0.1% formic acid, and 1% tert-butanol; and B—mixture isopropanol/buffer A (9/1, *v*/*v*).

### 4.10. Mass Spectrometry Conditions

Full-scan mass spectra over the *m*/*z* range 120–2000 were acquired in positive ion mode at resolution settings of 120,000. Data-dependent scans (ddMS) were carried out at Orbitrap resolution of 15,000 using isolation window of 2 *m*/*z*, intensity threshold 5.1 × 10^5^, dynamic exclusion 10 ppm with duration of 20 s, and fixed collision energy mode at 30%. The mass spectrometer operating parameters used in a positive ionization mode were as follows: spray voltage −3.5 kV; capillary temperature −300 °C; probe heater temperature −320 °C; sheath gas flow rate 30 units; auxiliary gas flow 12 units; sweep gas 3 units (units refer to arbitrary values set by the Orbitrap IQ-X tune software, version 4.3); and S-Lens RF level of 60.00. Nitrogen was used for sample nebulization and collision gas in the HCD cell. All derivatives were quantified using 5 ppm mass tolerance filters. Data acquisition and processing were carried out with XCalibur^®^ ver 4.7 software package (ThermoScientific Co., Waltham, MA, USA).

The identification of individual lipids and relative quantitative analysis were processed using LipidSearch 5.1 software package (ThermoScientific Co., Waltham, MA, USA).

### 4.11. Multivariate Data Analysis

To reveal the characteristic features of the lipid composition of the studied strains, quantitative information from the intensities of the respective chromatographic peaks of the identified compounds were used. These data sets were visualized by heatmap (Figure 2) of the extracts of the yeast’s biomass in the two solvents with ClustVis 2.0. [52]. The raw signals were automatically scaled by compounds to compensate for large concentration variation between them, and no transformation or sample-specific normalization was performed. All 126 compounds for overall view and top 50 metabolites ranked by their total intensity difference between classes were displayed to show the most contrasting pattern. The substances by rows are clustered based on their inter-group profile (Euclidean distance and Ward’s linkage).

## 5. Conclusions

This study demonstrates the biotechnological potential of the Antarctic yeasts *Dioszegia* sp. AL_105_ and *Bannozyma* sp. AL_104_ as sources of biologically active metabolites. Both strains were successfully cultivated under submerged conditions, with *Bannozyma* sp. AL_104_ growing faster and achieving a higher biomass production. The obtained extracts exhibited selective antiproliferative activity against malignant cell lines, moderate antibacterial activity, the inhibition of MRSA biofilm formation, and antioxidant potential. The biological activities were closely related to the chemical composition of the extracts. Methanolic extracts, particularly from *Dioszegia* sp. AL_105_, showed stronger antioxidant and antibiofilm effects, whereas the acetone extract of *Bannozyma* sp. AL_104_ demonstrated the highest cytotoxic activity. LC-MS and lipidomic analyses revealed the presence of bioactive metabolites, including CoQ_10_, sterol derivatives, apocarotenoids, phosphatidylethanolamines, and ceramides, which are known for their antioxidant and antiproliferative properties. The yeast extracts represent a complex mixture of different metabolites and their moderate activity could probably partially be due to the synergistic interaction between different components. The results in this study are the first screening of the activity of the extracts and reveal the bioactive potential of the extracts and represent a good basis for future pharmacological studies. The accumulation of new data on the biological activity of newly researched Antarctic extracts is a contribution to the world’s fundamental and applied knowledge for strains from genera *Dioszegia* and *Bannozyma*, division Basidiomycota.

## Figures and Tables

**Table 1:** Median inhibitory concentration of yeast extracts on cell lines from different origin.

Median Inhibitory Concentrations (IC_50_) for the Different Cell Lines [µg/mL ± SD]					
**Yeast Extract**	**CCL-1 ^a^**	**HUT-78 ^b^**	**MJ ^c^**	**T-24 ^d^**	**CAL-29 ^e^**
*Dioszegia* sp. AL_105_ methanol extract	>400	150.3 ± 11.6	220.1 ± 22.2	>400	257.9 ± 16.8
*Dioszegia* sp. AL_105_ acetone extract	>400	166.8 ± 10.7	181.1 ± 10.5	>400	>400
*Bannozyma* sp. AL_104_ methanol extract	>400	178.1 ± 16.9	209.8 ± 15.7	>400	278.8 ± 24.3
*Bannozyma* sp. AL_104_ acetone exrtract	>400	113.0 ± 8.2	>400	>400	>400

Legend: ^a^ normal murine fibroblast cells; ^b^ cutaneous T-cell lymphoma of the Sézary syndrome (leukaemic) type; ^c^ cutaneous T-cell lymphoma of the *Mycosis fungoides* (cutaneous) type; ^d,e^ urinary bladder transitional cell carcinoma cell lines.

**Table 2:** Minimal inhibitory concentrations (MICs) of *Bannozyma* sp. AL_104_ and *Dioszegia* sp. AL_105_ extracts on *S. aureus* and *E. coli*.

Extract	*Staphylococcus aureus* [mg/mL]	MRSA [mg/mL]	*Escherichia coli* [mg/mL]
*Bannozyma* sp. AL_104_	10	10	10
*Dioszegia* sp. AL_105_	5	5	10

**Table 3:** Inhibition of MRSA biofilm after exposure to *Bannozyma* sp. AL_104_ and *Dioszegia* sp. AL_105_ extracts.

Extracts	Concentration [mg/mL]			Untreated Control	Blank
	10	5	2.5		
*Bannozyma* sp. AL_104_	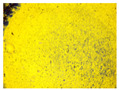	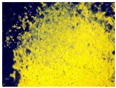	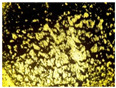	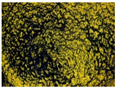	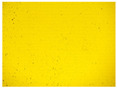
*Dioszegia* sp. AL_105_	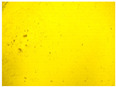	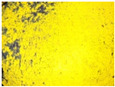	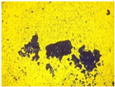	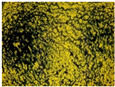	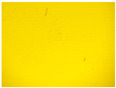
Graphical comparison	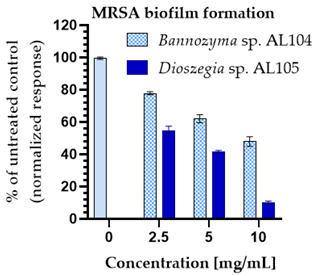				
Biofilm inhibition values		*Bannozyma* sp. AL_104_		*Dioszegia* sp. AL_105_	
	Concentration [mg/mL]	% of untreated control	SD	% of untreated control	SD
	0	100.00	±0.75	100.00	±0.75
	2.5	78.20	±0.93	54.68	±3.01
	5	62.36	±2.52	41.78	±0.89
	10	48.17	±3.10	10.30	±0.84

Legend: Untreated control—untreated bacterial cells; blank—empty samples (culture medium without extracts and bacterial suspension); SD—standard deviation.

**Table 4:** Antioxidant activity of yeast extracts.

MeOH Extract	HPSA		HRSA		NOSA
	µM QE/g LB	IC_50_, mg/mL	µM QE/g LB	IC_50_, mg/mL	IC_50_, mg/mL
*Dioszegia* sp. AL_105_	486.58 ± 5.81	0.64 ± 0.01	979.05 ± 56.11	1.10 ± 0.02	1.26 ± 0.16
*Bannozyma* sp. AL_104_	445.72 ± 12.05	0.71 ± 0.01	711.35 ± 100.54	1.28 ± 0.05	1.31 ± 0.08
Quercetin	-	0.069 ± 0.001	-	0.149 ± 0.009	0.065 ± 0.001

**Table 5:** Primary and secondary metabolites in the studied extracts.

N	Compounds	Distribution	Level of Confidence *
1	sucrose	1, 2, 4	D1
2	methylcitric/isocitric acid	1, 3	D1
3	isopropylmalic acid	1, 3	D1
4	mallic acid	2, 3	D1
5	azelaic acid	1, 2, 3, 4	D1
6	linoleic acid	3	D1
7	linolenic acid	3	D1
8	arginine	1, 3	D1
9	leucine	1, 3	D1
10	phenylalanin	1, 3	D1
11	dihydroactinidiolide	1, 2, 3	D2
12	3,5-dihydroxyergosta-7,22-dien-6-one	1, 2	D2
13	dehydroergosterol	1, 2, 3	D2
14	LysoPC (18:2) isomer 1	1, 2, 3	D2
15	LysoPC (16:0)	1, 2	D2
16	LysoPC (18:1) isomer 1	1, 2	D2
17	LysoPC (18:1) isomer 2	1, 2	D2
18	β-ionone	1, 2	D2
19	β-cyclocitral	2	D2
20	β-apo-13-carotenone	1, 2	D2

Legend: 1—*Dioszegia* sp. AL_105_ methanol extract, 2—*Dioszegia* sp. AL_105_ acetone extract, 3—*Bannozyma* sp. AL_104_ methanol extract, 4—*Bannozyma* sp. AL_104_ acetone extract. * Confidence level: D: Tentative identification based on libraries, model compounds, etc.; D1: relatively reliable evidence, match with any high-quality LC-MS/MS libraries validated for the particular class of metabolites; D2: relatively poor evidence, match with current LC-MS/MS libraries not yet validated exhaustively or by detailed comparison with homologues or other model compounds [19].

**Table 6:** Lipid composition of *Dioszegia* sp. AL_105_ and *Bannozyma* sp. AL_104_ extracts.

N	Lipid Group	Distribution	Level of Confidence *
1	Co(Q6)	3	D1
2	Co(Q7)	2, 3	D1
3	Co(Q8)	2, 3	D1
4	Co(Q6)	2	D1
5	Co(Q10)	1, 2, 3, 4	D1
6	Diglycerides (DG)	1, 2, 3, 4	D1
7	Triglycerides (TG)	1, 2, 3, 4	D1
8	Primary fatty acid amides (PFAA)	2, 3, 4	D1
9	Lysophosphatidylethanolamines (LPE)	2, 3, 4	D1
10	Phosphatidylcholines (PC)	2, 3, 4	D1
11	Phosphatidylethanolamines (PE)	3, 4	D1
12	Phosphatidylinositols (PI)	2	D1
13	Phosphatidylserines (PS)	2, 3	D1
14	Ceramides (Cer)	2, 4	D1

Legend: 1—*Dioszegia* sp. AL_105_ methanol extract, 2—*Dioszegia* sp. AL_105_ acetone extract, 3—*Bannozyma* sp. AL_104_ methanol extract, 4—*Bannozyma* sp. AL_104_ acetone extract. * Confidence level: D: Tentative identification based on libraries, model compounds, etc.; D1: relatively reliable evidence, match with any high-quality LC-MS/MS libraries validated for the particular class of metabolites [19].

## Data Availability

The original contributions presented in this study are included in the article/Supplementary Material. Further inquiries can be directed to the corresponding author.

## Supplementary Materials

The following supporting information can be downloaded at: https://www.mdpi.com/article/10.3390/molecules31142486/s1, Figure S1. Colony appearance and microscopic view: (A) *Dioszegia* sp. AL_105_; and (B) *Bannozyma* sp. AL_104_. Table S1. Cell growth dynamics and pH monitoring of culture by *Dioszegia* sp. AL_105_ and *Bannozyma* sp. AL_104_. Table S2. Two-way ANOVA for the antibiofilm activity of both yeast extracts. Table S3. Primary and secondary metabolites in the studied extracts.

## Abbreviations

The following abbreviations are used in this manuscript:

ROS	Reactive oxygen species
TG	Triacylglycerol
DG	Diacylglycerol
PE	Phosphatidylethanolamine
Co(Q10)	Coenzyme Q_10_
Co(Q7)	Coenzyme Q_7_
PS	Phosphatidylserine
Cer	Ceramide
Co(Q8)	Coenzyme Q_8_
LPE	Lysophosphatidylethanolamine
NCBI	Nacional Center for Biotechnology Information
LB	Lyophilized biomass
