# Supplementary material for: Unveiling the Chemical Composition and Biological Activity of Extracts from the Antarctic Yeast Dioszegia sp. AL105 and Bannozyma sp. AL104
Source: Molecules. 2026 Jul 16;31(14):2486. doi: 10.3390/molecules31142486 (PMC13415332; doi:10.3390/molecules31142486)
Supplement: Supplementary file 1 [file molecules-31-02486-s001.zip › Supplementary Table S2.pdf]

Supplementary Table S2. Two-way ANOVA for the antibiofilm activity of both yeast extracts.

**Šídák's multiple comparisons test**	**Mean Diff.**	**95.00% CI of diff.**	**Summary**	**Adjusted P Value**
Bannozyma sp. AL104				
0 vs. 2.5 mg/mL	21.68	9.552 to 33.81	**	0.0095
0 vs. 5 mg/mL	37.45	25.32 to 49.58	**	0.0019
0 vs. 10 mg/mL	51.56	39.43 to 63.69	***	0.0007
2.5 vs. 5 mg/mL	15.76	3.633 to 27.89	*	0.0239
2.5 vs. 10 mg/mL	29.88	17.75 to 42.01	**	0.0037
5 vs. 10 mg/mL	14.11	1.982 to 26.24	*	0.0327
Dioszegia sp. AL105				
0 vs. 2.5 mg/mL	45.08	32.95 to 57.21	**	0.0011
0 vs. 5 mg/mL	57.91	45.78 to 70.04	***	0.0005
0 vs. 10 mg/mL	89.22	77.09 to 101.4	***	0.0001
2.5 vs. 5 mg/mL	12.83	0.7047 to 24.97	*	0.0427
2.5 vs. 10 mg/mL	44.14	32.01 to 56.27	**	0.0012
5 vs. 10 mg/mL	31.31	19.18 to 43.44	**	0.0032
Bannozyma sp. AL104 vs. *Dioszegia* sp. AL105				
2.5	23.40	12.89 to 33.90	**	0.0051
5	20.47	9.965 to 30.97	**	0.0075
10	37.66	27.16 to 48.17	**	0.0012
