# Supplementary material for: Unveiling the Chemical Composition and Biological Activity of Extracts from the Antarctic Yeast Dioszegia sp. AL105 and Bannozyma sp. AL104
Source: Molecules. 2026 Jul 16;31(14):2486. doi: 10.3390/molecules31142486 (PMC13415332; doi:10.3390/molecules31142486)
Supplement: Supplementary file 1 [file molecules-31-02486-s001.zip › Supplementary Table S3.pdf]

Table S3. Primary and secondary metabolites in the studied extracts.

№	Identified/tentatively annotated compound	Molecular formula	Exact mass [M-H]⁻	Fragmentation pattern in (-) ESI-MS/MS	tR (min)	Δ ppm	Distri bution	Identification confidence level (Çiçek et al., 2024)
**Organic acids and sugars**								
1.	sucrose	$C_{12}H_{20}O_{11}$	341.1089	341.1089 (100), 179.0551 (49.3), 161.0443 (12.9), 143.0337 (13.8), 131.0336 (5.1), 119.0335 (35.1), 101.0229 (36.1), 89.0228 (94.0), 85.0278 (7.6), 71.0122 (66.3), 59.0122 (57.9)	0.92	-0.219	1,2,3	D1
2.	mallic acid	$C_4H_6O_5$	133.0143	133.0128 (17.1), 115.0020 (100), 71.0122 (91.6)	0.91	-10.875	2,4	D1
3.	citric/isocitric acid	$C_6H_8O_7$	191.0197	191.0089 (8.5), 173.0080 (3.5), 129.0179 (6.2), 111.0072 (100), 87.0071 (41.7), 73.0278 (1.9)	0.87	-4.114	4	D1
4.	citric/isocitric acid	$C_6H_8O_7$	191.0197	191.0089 (8.3), 129.0179 (5.8), 111.0072 (100), 87.0072 (41.1), 85.0279 (25.5)	1.06	-4.114	4	D1
5.	methylcitric/isocitric acid	$C_7H_{10}O_7$	205.0354	205.0346 (2.1), 173.0083 (1.2), 143.0337 (13.2), 115.387 (1.9), 111.0073 (100), 87.0072 (43.5), 83.0121 (0.8)	1.47	-3.979	3,4	D1
6.	isopropylmalic acid	$C_7H_{12}O_5$	175.0612	175.0601 (53.5), 157.0494 (10.9), 131.0700 (9.0), 129.0544 (0.8), 115.0385 (100), 113.0593 (40.1), 85.0643 (46.1)	3.30	-0.632	3,4	D1, Ricciuteli et al., 2019
**Fatty acids**								
7.	azelaic acid	$C_9H_{16}O_4$	187.0976	187.0967 (42.9), 169.0861 (2.4), 143.1068 (1.3), 125.0958 (100), 97.0643 (0.6), 83.0486 (0.5)	6.43	0.225	1,2,3,4	D1
8.	linoleic acid	$C_{18}H_{32}O_2$	279.2330	279.2324 (100), 261.2225 (0.5), 151.1234 (0.1), 118.1438 (0.1)	17.22	-0.693	4	D1
9.	linolenic acid	$C_{18}H_{30}O_2$	277.2173	277.2172 (100), 259.2064 (0.3), 233.2271 (0.4), 179.1935 (0.3), 119.1661 (0.3)	21.11	-0.518	4	D1
**№**	**Identified/tentatively annotated compound**	**Molecular formula**	**Exact mass [M+H]⁺**	**Fragmentation pattern in (+) ESI-MS/MS**	**tR (min)**	**Δ ppm**	**Distri bution**	**Identification confidence level (Çiçek et al., 2024)**
**aminoacids**								
10.	arginine	$C_6H_{14}O_2N_4$	175.1190	175.1189 (63.1), 158.0925 (14.1), 130.0976 (16.7), 116.0709 (41.5), 88.0768 (0.4), 70.0659	0.81	-0.241	3,4	D1

				(100), 60.0565 (56.6)				
11.	leucine	$C_9H_{13}O_2N$	132.1019	132.1020 (100), 116.0709 (0.3), 72.9915 (0.2)	0.90	0.793	3,4	D1
12.	phenylalanin	$C_9H_{11}O_2N$	166.0863	166.0863 (3.1), 149.0598 (1.1), 131.0492 (3.0), 121.0844 (5.3), 120.0809 (100), 107.0495 (1.9), 103.0546 (7.9), 93.0704 (1.9), 79.0549 (1.1)	1.68	0.270	3,4	D1
**Terpenes and sterols**								
13.	dihydroactinidiolide	$C_{11}H_{16}O_2$	181.1223	181.1222 (100), 163.1161 (13.9), 145.1011 (4.8), 135.1168 (19.5), 121.1013 (7.3), 109.1013 (1.8), 107.0859 (14.6), 93.0704 (6.9), 91.0546 (1.3), 81.0705 (3.2), 79.0549 (2.5)	10.12	-0.808	2,3,4	D1 Rehman et al., 2022
14.	3,5-dihydroxyergosta-7,22-dien-6-one	$C_{28}H_{42}O_3$	427.3207	427.3200 (27.1), 409.3095 (100), 391.2996 (9.3), 339.2310 (2.2), 325.2156 (1.0), 285.1841 (2.5), 267.1738 (13.4), 249.2214 (5.2), 231.2107 (2.6), 191.1067 (38.6), 187.1117 (32.6), 175.1116 (15.4), 173.0961 (35.8), 159.1170 (3.4), 145.1013 (9.3), 145.1013 (9.3), 105.0704 (6.1), 95.0859 (6.8), 83.0862 (8.7), 69.0706 (31.8)	20.20	-1.548	2,3	D1 Le et al., 2023
15.	dehydroergosterol	$C_{28}H_{42}O$	395.3308	395.3303 (100), 377.3202 (19.9), 335.2715 (1.1), 325.2531 (3.4), 311.2362 (16.5), 293.2258 (8.1), 269.1898 (9.1), 251.1802 (10.3), 229.1898 (2.8), 211.1482 (7.1), 199.1486 (5.6), 171.1168 (3.9), 157.0121 (5.6), 145.1012 (3.7), 125.1327 (28.9), 107.0858 (5.1), 83.0862 (28.9), 81.0705 (8.9), 69.0707 (95.9)	20.73	-1.398	2,3,4	D1
**Lysophosphatidylcholines (LysoPCs)**								
16.	LysoPC (18:2) isomer 1	$C_{26}H_{50}O_7NP$	520.3398	520.3392 (35.4), 502.3281 (1.2), 337.2711 (0.3), 184.0733 (100), 163.0138 (0.2), 125.0000 (21.1), 98.9846 (3.1), 86.0970 (29.7), 60.0816 (5.8)	14.93	-1.030	2,3,4	D1
17.	LysoPC (18:2) isomer 1	$C_{26}H_{50}O_7NP$	520.3398	520.3390 (93.1), 502.3293 (5.2), 337.2720 (1.4), 184.0732 (91.9), 124.9999 (27.9), 104.1074 (100), 86.0970 (48.8), 98.9844 (3.4), 71.0737 (6.9), 60.0816 (16.9)	15.26	-1.491	2,3,4	D1
18.	LysoPC (16:0)	$C_{24}H_{50}O_7NP$	496.3398	496.3393 (100), 478.3291 (7.5), 347.3122 0.5), 313.2727 (2.2), 184.0733 (84.7), 124.9999 (27.3), 104.1074 (95.8), 86.0970 (44.8), 60.0816 (17.1)	16.00	-1.019	2,3,4	D1

19.	LysoPC (18:1) isomer 1	$C_{26}H_{52}O_7NP$	522.3554	522.3549 (31.9), 504.3476 (1.3), 245.0716 (0.2), 184.0732 (100), 124.9999 (20.1), 104.1075 (2.7), 98.9848 (3.5), 86.0970 (29.0), 60.0816 (5.8)	16.17	-1.064	2,3,4	D1 Shen et al., 2016 Sorgn et al., 2022 Murphy and Axels 2010
20.	LysoPC (18:1) isomer 2	$C_{26}H_{52}O_7NP$	522.3554	522.3549 (86.2), 504.3444 (5.1), 455.2704 (0.5), 184.0732 (88.8), 163.0156 (0.6), 124.9999 (26 8), 104.1073 (100), 98.9848 (4.4), 86.0970 (55.8), 71.0736 (8.6), 60.0816 (20.6)	16.57	-1.064	2,3,4	D1
**Apocarotenoids**								
21.	β-ionone	$C_{13}H_{20}O$	193.1587	193.1585 (100), 175.1481 (3.2), 149.1325 (13.0), 137.0960 (11.6), 135.1168 (33.0), 133.1012 (18.9), 123.1169 (22.3), 109.0651 (82.2), 107.0858 (25.7), 97.0704 (10.0), 95.0496 (22.3), 93.0704 21.4), 91.0944 (3.6), 79.0549 (5.1), 71.0499 (12.9), 69.0706 (21.7), 67.0551 (5.6)	15.74	-0.786	2,3	D2
22.	β-cyclocitral	$C_{10}H_{16}O$	153.1274	153.1273 (52.1), 135.1168 (21.2), 125.0964 (2.9), 109.1015 (32.6), 107.0859 (53.3), 97.0653 (54.2), 93.0704 (100), 91.0547 (13.0), 79.0549 (17.22), 69.0707 (12.8), 65.0394 (0.9)	16.79	-0.468	2	D2
23.	β-apo-13-carotenone	$C_{18}H_{26}O$	259.2056	259.2054 4.7), 241.1947 (5.5), 215.1793 (3.8), 185.1328 (4.8), 175.1480 (32.9), 159.1163 (10.4), 145.1010 (7.6), 133.1012 (9.7), 123.1167 (10.8), 119.0857 (19.5), 109.0651 (6.9), 107.0861 (6.9), 97.0651 (9.6), 5.0860 (6.1), 93.0703 (4.8), 91.0546 (1.9), 79.0548 (1.8), 71.0862 (1.5), 69..1278 (4.3), 67.0549 (1.7)	21.34	-1.011	2,3	D2

Legend: 1- *Bannozyma* sp. aceton extracts; 2- *Dioszegia* sp. aceton exstracts; 3- *Dioszegia* sp. methanol extracts; 4- *Bannozyma* sp. methanol extracts
