# Supplementary figures and images for: Unveiling the Chemical Composition and Biological Activity of Extracts from the Antarctic Yeast Dioszegia sp. AL105 and Bannozyma sp. AL104
Source: Molecules. 2026 Jul 16;31(14):2486. doi: 10.3390/molecules31142486 (PMC13415332; doi:10.3390/molecules31142486)

**Figure S1.** Colony appearance and microscopic view. (A) *Dioszegia* sp. AL105; (B) *Bannozya* sp. AL104.

A)

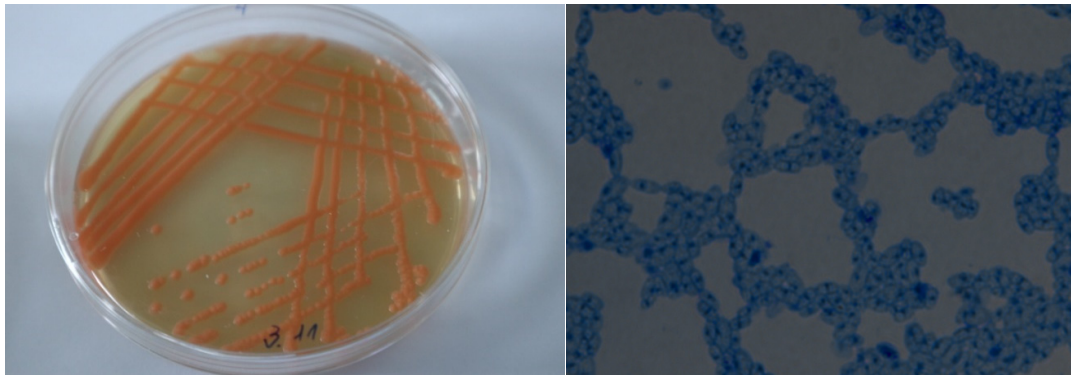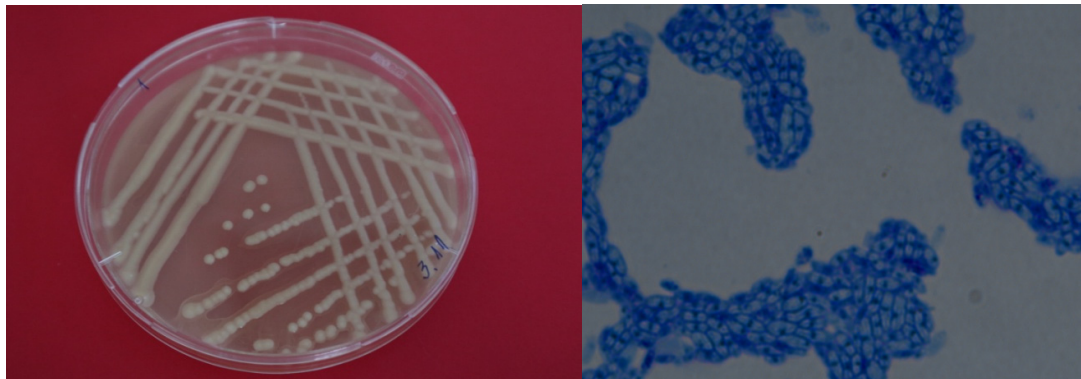

B)

Supplement: Supplementary file 1 [file molecules-31-02486-s001.zip › Supplementary Figure S1.pdf]
